# A Rare Case of Mediterranean Spotted Fever and Encephalitis

**DOI:** 10.1155/2016/2421540

**Published:** 2016-12-07

**Authors:** Raquel Sousa Almeida, Petra M. Pego, Maria João Pinto, João Matos Costa

**Affiliations:** 3rd Department of Internal Medicine, Hospital Distrital de Santarém, Santarém, Portugal

## Abstract

Mediterranean spotted fever is a tick-borne zoonotic disease caused by* Rickettsia conorii*. It is transmitted by the dog tick* Rhipicephalus sanguineus*. It usually presents as a benign self-limited disease characterized by a skin rash, high fever, and, sometimes, a characteristic ulcer at the tick bite site called* tache noir*. The course of this disease is usually benign, although severe manifestations have been previously described, mainly in adults. Neurological manifestations are very unusual. We present a case of Mediterranean spotted fever with encephalitis to highlight the importance of clinical suspicion, mainly in endemic areas, the potential severity of this disease, and the need of early initiation of therapy in order to prevent severe complications.

## 1. Introduction

Mediterranean spotted fever (MSF) is an emerging zoonosis caused by* Rickettsia conorii*, a member of the spotted fever group of rickettsiae [1].* Rhipicephalus sanguineus* (dog tick) is the only recognized tick vector of rickettsiae identified in Portugal. Most of the cases occur during summer, during the period from July to September [2]. MSF is usually a benign and self-limited disease, characterized by skin rash, high fever, and a characteristic ulcer at the tick bite site called* tache noir*. Severe presentations are unusual but have been increasingly reported [3, 4]. Diagnosis is based on epidemiological, clinical, and laboratory criteria. The reference method is immunofluorescence which allows for the detection of IgM and IgG in the acute and convalescent sera [5]. Doxycycline (200 mg/day during 7–14 days, depending on the clinical course) is the drug of choice for the treatment of MSF [6].

## 2. Case Presentation

**Figure 1:**
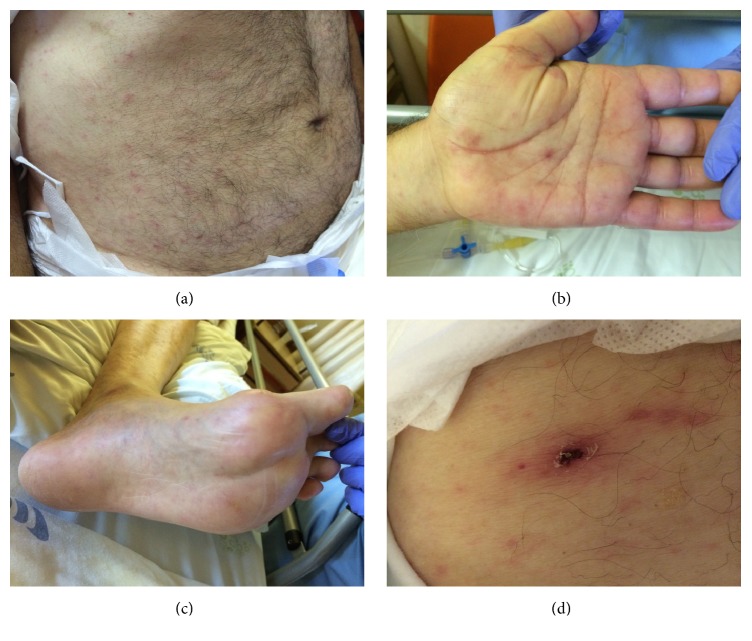
Maculopapular disseminated rash (a), including the palms of the hands (b) and the soles of the feet (c) and a dark brown inoculation eschar (*tache noir*) in the left inguinal region (d).

A 79-year-old male presented to the emergency department in August with high fever, headache, myalgia, nausea, and vomiting since the past six days and also with confusion and left hemiparesis since earlier that day. He had a previous history of arterial hypertension, diabetes mellitus, and chronic sinusitis. He lived in a rural area and had regular contact with dogs. On physical examination he was febrile (38,9°C), his blood pressure was 165/80 mmHg, and his pulse was regular, 120 beats per minute. He had a disseminated maculopapular rash, including the palms of the hands and the soles of the feet (Figures 1(a)–1(c)). A dark crusted lesion with a diameter of approximately 50 mm consistent with* tache noir* was noticed in the left inguinal region (Figure 1(d)). Neurological examination revealed decreased level of consciousness (Glasgow Coma Score of 10), left hemiparesis, and left hypoesthesia, including the face. Global aphasia and labial commissure deviation to the right side were also noted. The rest of the physical examination was normal, including absence of meningeal signs and normal flexor plantar reflexes. His haemoglobin level was 12.2 g/dL, his white blood cell count was 8700 cells/*μ*L (88,5% neutrophils, 8% lymphocytes, and 3% monocytes), and his platelet count was 101 000 platelets/*μ*L. His C-reactive protein level was 20,70 mg/dL. The remainder laboratory evaluation showed hyperglycaemia (167 g/dL), acute renal failure (2.2 mg/dL creatinine), and elevated liver enzymes (140 U/L aspartate aminotransferase, 136 alanine transferase U/L). Urinalysis and chest radiograph were unremarkable.

Cerebral computerized tomography (CT) scans were performed at admission and 48 hours later, both with no abnormalities. A lumbar puncture was performed on the first day of admission and cerebral spinal fluid (CSF) analysis revealed moderately elevated protein, normal glucose level, and pleocytosis (48 cells/*μ*L) with polymorphonuclear predominance. Pending results of diagnostic studies, an empirical regimen of acyclovir, ceftriaxone, and doxycycline was started, with a slight neurological improvement in 24 hours. However, later that day, the patient presented with tonic-clonic seizures that ceased with intravenous diazepam and was transferred to the intensive care unit. The EEG examination was not performed as it was not available at the site and the patient was not clinically stable to be transferred. Microbiological cultures and PCR for herpes simplex virus in the CSF were negative. Serology by indirect immunofluorescence assay showed elevated IgM antibodies titer (≥32; negative if < 32, positive if ≥ 32) for* Rickettsia conorii*, with nonelevated IgG (<64; negative result if < 64, suspicious if = 64, and positive if ≥ 128). After the third day in doxycycline therapy, there was a gradual clinical improvement, with progressive normalization of inflammatory markers, renal function, and liver enzymes. After eight days of doxycycline therapy, neurological examination was normal. He was discharged home with normal laboratory tests and with no neurological sequelae. In a new sample, taken 15 days after the initial presentation, the IgG antibodies for* Rickettsia conorii* were positive (≥128). The patient has been followed in our clinic, with no episodes of seizures, neurological deficits, or other symptoms.

## 3. Discussion

In this patient, the first signs of were typical. The episode occurred during summer, with characteristic symptoms of fever, rash, and* tache noir* lesion and the acute and convalescent serological tests were confirmatory. However, it was complicated by neurological manifestations, acute renal failure, acute hepatic failure, and thrombocytopenia. The course of MSF is usually benign; however severe manifestations have been previously described. Advanced age, chronic alcoholism, immunocompromised status, glucose-6-phosphate dehydrogenase deficiency, prior prescription of inappropriate antimicrobial therapy, delay in treatment, and diabetes are risk factors for more severe presentations which can lead to a fatal outcome [2, 7]. The pathogenesis of MSF complications results from* Rickettsia* invasion and its multiplication in vascular endothelial cells, resulting in widespread vasculitis of capillaries, arterioles, and small arteries [7]. Renal impairment has been frequently described as a consequence of severe MSF [8]. In this patient, the delay in seeking medical attention and treatment, advanced age, and previous diabetes mellitus history probably accounted for the severity of the manifestations. Assuming the possible cross-reactions with other emerging* Rickettsia* of the spotted fever group, the use of standard serological tests for diagnosis is a limitation in our observation. Only five cases of adults with encephalitis related to* Rickettsia conorii* infection diagnosed by IFA and with CSF analysis description are described in the literature. The majority (three in five) of the cases reported pleocytosis in the CSF. Elevated protein levels were found in three cases. The glucose levels were normal in one case, slightly elevated in two, and decreased in one [1, 2, 9]. Of the four who survived, only one patient recovered without sequelae [1, 2, 9]. Although systematic pharmacokinetic studies are lacking for the concentration of doxycycline in CSF against* Rickettsia conorii*, a study on neuroborreliosis showed that daily doses of 200 mg of doxycycline produce a CSF concentration close to the MICs for susceptible bacteria, both in the absence and in the presence of meningeal inflammation [10]. Doxycycline is the most effective antibiotic against* Rickettsia conorii*, with a MIC of 0,06 *μ*g/mL [11]. Fluoroquinolones may be considered a safe alternative to tetracyclines for the treatment of rickettsial diseases. However, the potential toxicity of doxycycline and fluoroquinolones contraindicates their use during pregnancy and childhood [12]. Clarithromycin is considered a valid alternative in patients with hypersensitivity to tetracyclines, in pregnant women and in children [13, 14].

## 4. Conclusion

Rickettsiosis is emerging infectious disease, which usually has a benign course. Nonetheless, clinical awareness is crucial, mainly in endemic areas, as they may present with severe life-threatening complications, such as encephalitis. This case report highlights the importance of early initiation of therapy in order to prevent these severe complications.
